# One Hundred Consecutive Cases of Skin Disease

**Published:** 1891-07

**Authors:** M. B. Hutchins

**Affiliations:** Atlanta, Ga.; Lecturer on Diseases of the Skin, Atlanta Medical College


### ONE HUNDRED CONSECUTIVE CASES OF SKIN DISEASE.

### VI.

By M. B. HUTCHINS, M. D.,

Lecturer on Diseases of the Skin, Atlanta Medical College.

### SYPHILIS.

It is necessary in making the diagnosis of syphilis to depend upon the appearances of the skin lesions and to have recourse to the history only as confirmative evidence, if a history of infection is possible of attainment. Patients with syphilis frequently do not know they have it, or, knowing, think it their imperative duty to deny, and mislead the doctor if possible. Nearly every one of the cases to be described were diagnosed without regard for the "history," and the diagnosis, where positive, was fully confirmed by results of "specific" treatment together with, in some cases, confessions of the patient.

Of the one hundred cases of skin disease under description there were eight (or, including one with only subjective skin symptoms, of which the diagnosis was doubtful, nine) cases of syphilis. As the diagnostic points may be of general interest, I give the symptoms in detail in most of the cases.

First—lady, age forty-nine. There were groups of crusted, slightly infiltrated lesions on occiput and end of nose. Similar lesions on lips and fissures in each oral angle. Removal of crusts exposed raw, slightly bleeding and purulent ulcerative surface. The only "history" was of sore throat and rheumatic attacks in one leg a few months before. The husband was presumed (as circumstances indicated) to have had the disease, and to have infected this patient. (This information came out after the diagnosis was communicated.) For prudential reasons the patient received treatment at a famous resort for such cases, and is said to have returned free of the eruption.

Case two—female, age sixty. Gummatous, round, clear cut, silver quarter sized ulcer on lower leg.

Locally, used:

℞. Ungt. Hg., ʒii.

Ungt. O. Z., ʒvi.

M. Sig.—Keep applied, which aided in causing the ulcer to heal rapidly. Internally the ordinary mixed treatment was given. Probable infection from syphilitic grandchild.

Case three—female, age sixty-eight. Husband "died before the war." "Eruption" sixteen years before the present time, and ulceration of the throat persisting for nearly a year.

When seen, there weie present on nose and upper lip brownish crusts covering superficial ulceration, which secreted thin pus.

In naso-facial region small, crusted, discrete lesions with slight ulceration, and on left cheek an irregular, pigmented scar. From left shoulder to meso-dorsal region irregular, smooth scars, as after a burn, terminating below in a large plaque of grouped crusts, removal of which exposed superficial, clean-cut, roundish ulcerations. (The scarring above and ulceration below showed, quite typically, the progress of the serpigmous siphilitic ulceration.) A deep, pigmented scar, as large as the palm, situated near the tendon achillis, of right leg, marked the site of an ulcer which persisted for five years. "History," save of course of lesions, negative. Ungt. hydrarg. and zinc oxide, as for case two, was used locally, and "mixed treatment" (of which the following is an example):

℞. Hg. bichlor., gr. i.

Potass. iodat., ʒi–ʒiii.

Tinct. cinchon. c.,

Tinct. gent. c., aa ℥ii.

M. Sig.—ʒi in half glass of water after meals, was given internally.

Lesions had practically disappeared at end of a month, after which the patient was not seen.

Case four—male, age twenty-one. Chancre of cheek.* Just in front of tragus of right ear was a circular, dome-like elevation the size of a silver quarter, summit crusted as the result of applications. Removal of crust showed appearance as if summit of lesion had been smoothly cut off, a circular erosion the size of a dime, granular, nutmeg looking, perforations filled with thin pus, the remainder of the erosion "raw" looking, and showing just a little moisture. There was a group of enlarged submaxillary lymphatics on corresponding side. Two weeks later the throat was very red, but returned to normal under use of simple alum solution, as gargle. The local treatment as used on the other cases for late sypilitic ulcerations was tried, as an experiment, on the chancre, but it remained, altogether, about six weeks.

*Reported before the Atlanta Society of Medicine.

Slight macular eruption occurred on abdomen and back about four weeks from first appearance of chancre. This eruption was most developed after being present two weeks, and its whole duration was about four weeks. General adenopathy was not present, only one enlarged lymphatic, in back of neck, being perceptible. The enlarged lymphatics were reduced upon the healing of the chancre. Rheumatism in left shoulder was the only other symptom, save, perhaps, occasional headache. Treatment was with simple tonics and remedies for constipation until the eruption appeared ; then one-thirty-second of a grain of bichloride of mercury in a teaspoonful of compound tinctures of cinchona and gentian was given. Four months after occurrence of chancre there was a "mucous patch" beneath the tongue. Hydrarg. was doubled in dose and

℞. Hg. bichlor., gr. i.
Mucilag. Acac.,
Aquae, aa ℥ss, was applied locally to the ulcer. Save one relapse of "mucous patches," which rapidly healed, the patient had no further trouble.

After the diagnosis was made, the patient told me that his working companion had a long-standing case of syphilis, with numerous mucous patches in the mouth. One day, in play, he tried to bite patient's ear, and thus probably innoculated a slight scratch where the initial lesion afterward occurred.

The next case was that of a young man of nineteen, previously under treatment for acne. There was a typical penile chancre, and the macular eruption on the lower part of abdomen and upper thighs. Was given pill of protoiodide of mercury, gr. 1-5, t. i. d. Epitrochlear and cervical glands were typically enlarged. After disappearance of the eruption he had no further trouble. The patient's acne rapidly disappeared just after treatment was begun for the new disease. It is still an open question whether the syphilis, the "specifiic treatment" or the acne treatment caused this rapid disappearance. However, the acne recurred later while patient neglected local treatment.

The next two cases were, and still are, uncertain, as the diagnosis, so far as made, in one was after an examination by lamp-light of a leg affected with a few ulcers and an iodoform eczema, and the other case was one in which symptoms of dermatalgia, and some scars, led a New York dermatologist to refer the patient back to me with the diagnosis of "probable sypilitic congestion of roots of spinal nerves." The treatment did not confirm diagnosis.

The next case shows the protean form of the disease in a marked degree. Female, age forty-two, referred for diagnosis. Between metacarpals, dorsal surface, of thumb and index finger an oblong infiltrated patch of purplish color, about the border of which were a few dime-sized, hard, roundish tubercles, with depressed and slightly scaly center. Color of patch removable under pressure. Small, oblong patch in similar situation on left hand corresponded to that on right. There was a group of waxy looking papular lesions on point of left shoulder, pea to small finger nail in size, surface thinly crusted (from scratching) and an oblong group of declining lesions (papules) back of left arm near border of axilla. The papules were from purplish, at beginning, to fawn color as they declined, and left stains of latter shade. When moist from perspiration the eruption itched, and scratching was followed by scaling and then crusts. Has had eleven children, all of whom died at the ages of from a few weeks to a few months, save the eighth, ninth and eleventh. The patient appeared in good general health.

The last of the series was a beautiful case for diagnosis. Patient male, age thirty-eight. Duration of disease three years, symptoms always as when this "history" was written. One large patch on left palm and smaller ones on palmer surface of, or between the fingers of, both hands, one or two tending to creep over on dorsal surface of fingers The patch is thick, dry, scaly, and grayish in color, and tends to superficial fissuring. Close inspection shows this patch to be composed of separate, circular lesions the size of a half-dime. Disease has progressed towards ulnar edge of palm and undergone resolution on radial side. Whole eruption dry in character and belongs to the type

"papulo-squamous syphilide." The finger lesions are half-dime sized, dry and scaly, roundish, well-defined, when not confluent, and in rows or irregular lines, the whole process showing tendency to (and history proves) spreading in a serpigmous manner.

One lesion, on a finger, had the sometimes typical scaly collar. No "specific history" nor presence of other lesions.

Treatment. Rub away thickened epidermis, and

℞. Emplastrum hydrarg.

M. Sig.—Apply in daytime.

℞. Ungt. hydrarg.

M. Sig.—Apply, and wear gloves, at night.

℞. Hg. bichlor., gr. i.

Kalii Iodat., ℥i.

T. Nucis Vom., ʒii.

Aquae ad. ℥iv.

M. Sig.—ʒi in half a glass of water after meals.

Patient returned to his home in another State and has not since reported.

Trichophytosis, etc., next article.

1½ *Edgewood Avenue.*

---

AMERICAN MEDICAL ASSOCIATION.—The session in Washington, D. C., May 5–8, was a decided success. Officers for 1891–2 are: President, Dr. H. O. Marcy, of Boston; Vice-Presidents, Drs. Willis P. King, of Missouri; Henry Palmer, of Wisconsin; W. E. B. Davis, of Birmingham, Ala.; W. E. Taylor, of San Francisco, Cal.; Treasurer, Dr. Richard. J. Dunglison, of Philadelphia, Pa.; Secretary, Dr. Wm. B. Atkinson, of Philadelphia, Pa. Place of Meeting 1892, etc., Detroit, Mich., first Tuesday in June, 1892. Dr. H. O. Walker, Chairman of Local Committee of Arrangements. Among the many elegant receptions, that given by Dr. and Mrs. Wm. A. Hammand, in their palatial residence, "Belcourt," will long be held in special memory for its magnificence and hospitality.—*Va. Medical Monthly.*

